# Fabrication of Ordered Porous Polyimide Films Templated by (AB)_m_ Type of Diblock Copolymer

**DOI:** 10.3390/polym15030635

**Published:** 2023-01-26

**Authors:** Mengwei Huo, Yibo Wang, Xinbo Wang

**Affiliations:** School of Materials Science and Engineering, Harbin Institute of Technology, Weihai 264209, China

**Keywords:** polyimide-*b*-polystyrene, net-like morphology, porous PI films, solvent uptake

## Abstract

Ordered porous polyimide films were fabricated from (AB)_m_ type of diblock copolymer polyimide-*b*-polystyrene (PI-*b*-PS). An increase in PS volume fraction (*f ^V^_PS_*) is beneficial to decrease the relative dielectric constants and water contact angles of the obtained porous PI films: the relative dielectric constant decreases to 1.89 and water contact angle decreases to 43° when *f ^V^_PS_* is 0.36, indicating porous PI films can be used as low-κ material and have good wettability. The solvent uptake of the porous PI films increases with increasing *f ^V^_PS_*. A net-like morphology appears when *f ^V^_PS_* reaches 0.36. The net-like porous PI film reaches equilibrium uptake of water within approximately 2.5 h, with a final equilibrium uptake ratio of 17.5%, and reaches equilibrium uptake of toluene within approximately 8 h, with a final equilibrium uptake ratio of 15.4%, displaying the highest and fastest solvent uptake compared with other microstructured porous PI films, which is ascribed to the specific characteristic of the interconnecting porous channels derived from the self-assembly of PI-*b*-PS with *f ^V^_PS_* 0.36. Introducing thermally degradable segments to PI-based block copolymer is an effective method to prepare porous PI films and can enhance some of their properties.

## 1. Introduction

As a high-performance polymer, aromatic polyimide (PI) exhibits excellent mechanical property, thermal stability, chemical resistance and electrical property. Currently, porous PI films, a functional PI material, have received more extensive attention for their wide applications in photoelectric technology, photonics, separation and biotechnology, etc. [1,2,3]. Many methods have been adopted to fabricate porous PI films, including porogen [4,5,6], block copolymer [7,8], breath figure [9,10], phase inversion [11], etc. 

Among these methods, self-assembly of block copolymers is a promising approach, by which the labile block is removed and an ordered porous structure forms in the remaining matrix. Generally, polypropylene oxide [12], polypropylene glycol [13], polyethylene glycol [14], polycaprolactone [15] and polymethylmethacrylate [16] have been used as the etchable block in this approach. It is known that block copolymers can self-assemble into various ordered nanostructures such as sphere, cylinder, gyroid, and lamellae [17]. Moreover, the nanostructures and the microdomain sizes of block copolymers can be easily finetuned by changing their volume fraction and molecular weight. Studies have shown that nanoporous polymers with a well-ordered pore structure and tunable mass transport characteristic are of major importance in frontier high-tech fields [18]. Therefore, preparation of nanopores from block copolymer templates has attracted wide attention. This method involves a removing process for a labile block in which thermally removing process is most commonly used. Thus, the thermally removable block is decomposed, and a nanoporous structure forms in the PI matrix.

Compared with other thermally removable blocks, Hedrick and coworkers [19] found PS provides a slow and mild decomposition condition in PS-*b*-PI-*b*-PS triblock copolymer, which can avoid rapid depolymerization and excessive plasticization of the polyimide from the degradation of by-products upon thermolysis of the labile PS block, along with the expected reduction in mass density. Hwang and coworkers’ research [20] showed that the annealing treatment is assumed to be essential to prepare the optimized PS-*b*-PI-*b*-PS triblock copolymer for the foamed structure. In addition, by tuning the molecular weight of PS-*b*-PI-*b*-PS triblock copolymers, the volume fraction of PS and the size of the final pores can be easily changed [21]. 

All the block copolymers used in the aforementioned research works are ABA-type triblock copolymers; AB type of PI-based block copolymers are rarely used as templates to fabricate porous PI films. In this study, we aim to demonstrate a fabricating route through the self-assembly of block copolymers for the preparation of ordered porous PI films. Amino endcapped polystyrene (PS) was first prepared by telomerization using a disulfide, and then anhydride endcapped polyamic acid (PAA) was connected covalently by PS, forming (AB)_m_ type of diblock copolymer PAA-*b*-PS. After treatment at 350 °C, PAA block was transformed to PI, accompanied by the decomposition of PS, resulting in the formation of porous PI films. The absorption behavior and dielectric property of different porous PI films were examined and analyzed. 

## 2. Materials and Methods

### 2.1. Materials

Azodiisobutyronitrile (99.5%, AIBN), styrene (≥98%, St) and anhydrous N,N-dimethylacetamide (≥99%, DMAc) were purchased from Sinopharm Chemical Reagent Co., Ltd. Bis(4-aminophenyl) disulfide (98%, BAPD) was purchased from Aldrich. Pyromellitic dianhydride (≥99%, PMDA) and 4,4′-diaminodiphenyl ether (≥99%, ODA) were purchased from Forsman Scientific (Beijing) Co., Ltd. Styrene was distilled under reduced pressure before use. PMDA was dried at 125 °C in a vacuum for 24 h before use. All other reagents were used without further purification. 

### 2.2. Preparation of Amino Endcapped PS

**Scheme 1:**
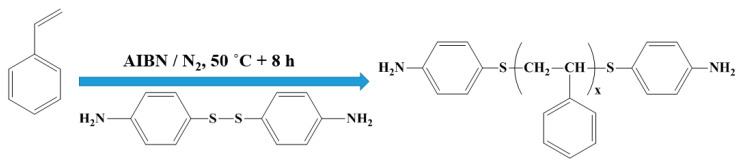
Preparation of amino endcapped PS.

Scheme 1 presents a few modifications of the preparation process according to a previous reference [22]. Typically, St (100 g, 0.96 mol), BAPD (3.7 g, 0.015 mol), and AIBN (0.05 g, 0.0003 mol) were added into a 200 ml Schlenk flask with a magnetic stirrer, then the mixture was stirred until a homogeneous solution formed. After degassing by three freeze–evacuate–thaw cycles, the flask was sealed under high-purity nitrogen, the polymerization was carried out at 50 °C for 8 h. The obtained viscous mixture was poured into a 10-fold volume of ethanol, and the precipitate was collected by filtration. After three times of dissolution–precipitation, the precipitate was dried in a vacuum at 60 °C overnight. The powdered NH_2_-PS-NH_2_ was acquired.

### 2.3. Preparation of PAA-b-PS 

**Scheme 2:**
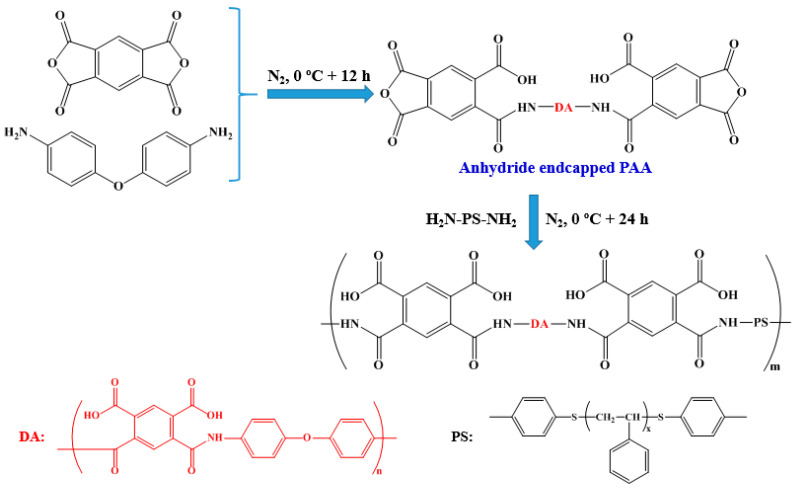
Schematic procedure to prepare PAA-*b*-PS copolymer via a chemical combination of polyamic acid and modified PS.

Scheme 2 shows a schematic diagram of the procedure to synthesize PAA-*b*-PS. ODA and DMAc were placed in a 250 ml three-necked flask, and the solution was stirred at 0 °C for 30 min under a nitrogen atmosphere. PMDA was then added in several batches, and the final solution concentration was controlled at 10 wt%. The mixture was continuously stirred at 0 °C for another 12 h under a nitrogen atmosphere. Three anhydride endcapped PAAs from different molar ratios of PMDA/ODA were prepared. Moderate amounts of DMAc solutions containing NH_2_-PS-NH_2_ (10 wt%) were introduced to the obtained three PAAs solutions, in which the mole ratio of amino and anhydride was 1:1; each mixture was stirred at 0 °C for 24 h under a nitrogen atmosphere and stored in a refrigerator prior to use. In this study, the obtained PAA-*b*-PSs, if not explained specifically, were all (AB)_m_ type of diblock copolymers. 

### 2.4. Preparation of Porous Polyimide Films

The above three DMAc solutions of PAA-*b*-PS were casted on a glass substrate. The solvent was removed in a vacuum oven at 50 °C for 8 h. The dried PAA-*b*-PS films were thermally treated under a flowing nitrogen atmosphere in sequential procedure: heating up to 100 °C in 30 min + 100 °C for 1 h → heating up to 200 °C in 30 min + 200 °C for 1 h → heating up to 350 °C in 50 min + 350 °C for 4 h. Finally, the porous PI films were formed. 

### 2.5. Measurements

The weight-average molecular weight ($\bar{M_{w}})$, number-average molecular weight ($\bar{M_{n}})$ and molecular weight distribution ratio *Đ* ($\bar{M_{w}}/\;\bar{M_{n}})$ of the synthesized polymers were determined via gel permeation chromatography (GPC) (Waters 1515, Waters Corporation, USA) equipped with two columns (Styragel HR 3 DMF 7.8 × 300mm Column and Styragel HR 4 DMF 7.8 × 300mm Column) at 35 °C with a flow rate of 1 mL/min. N,N-dimethylformamide (DMF) was used as the mobile phase. The elution time was detected by Waters 2414 Refractive Index Detector. The molecular weight and polydispersity index were calculated by the Water BreezeTM software. Calibration was performed using narrow distribution polystyrene standards (Polymer Laboratories). 

The molecular weight of PAA-*b*-PS copolymer was calculated using the Carothers equation:

(1) $$\bar{X_{n}}=\left(1+r\right)/\left(1+r-2rp\right)$$

where r represents stoichiometric functional groups ratio of initial two monomers (r ≤ 1); p represents the extent of reaction. Here, *p* value is close to 1 because of high reactivity between dianhydride and diamine, then Equation (1) can be simplified as follows:

(2) $$\bar{X_{n}}=\left(1+r\right)/\left(1-r\right)$$

Fourier transform infrared spectroscopy (FTIR) spectra were recorded at a resolution of 1cm^−1^ with a Nicolet 380 spectrometer. 

The surface morphologies were recorded via field-emission scanning electron microscopy (FESEM) on a JEOL JSM-6700F using accelerating voltages of 1.5 to 3 keV. The thermogravimetric analysis (TGA) was performed using a TG209F3 instrument thermal analyzer system (Netzsch) at the heating rate of 10 °C/min under the N_2_ condition with a flow rate of 50 mL/min. 

The relative dielectric constant of the porous films was determined by the Maxwell–Garnett approximation [23] as Equation (3):

(3) $$f_{1}\left(\frac{\varepsilon_{1}-\varepsilon }{\varepsilon_{1}+2\varepsilon }\right)+f_{2}\left(\frac{\varepsilon_{2}-\varepsilon }{\varepsilon_{2}+2\varepsilon }\right)=0\;$$

where *f*_1_ and *f*_2_ are volume factions of pores (air) and PI matrix, respectively; *ε_1_* and *ε_2_* are relative dielectric constants of air and pure PI, respectively; ε is the relative dielectric constant of porous PI films. Since the values of *ε_1_* and *ε_2_* are 1 and 3.3 (10^3^ Hz) [24], respectively, Equation (3) can be simplified as Equation (4):

(4) $$\varepsilon =\frac{-\left(6.9f_{1}-5.6\right)\pm \sqrt{{\left(6.9f_{1}-5.6\right)}^{2}+26.4}}{4}$$

The liquid uptake test is operated as follows: each film with the weight of 1 g is immersed in liquid at 25 ± 1 °C. The soaked film is taken out from the liquid every 1 h and the liquid on the surface is wiped off; then the weight of each soaked film is recorded. The liquid uptake was calculated using Equation (5):

(5) $$\mathrm{uptake}=\frac{W_{wet}-W_{dry}}{W_{dry}}\times 100\%$$

where *W_wet_* and *W_dry_* are the weights of the liquid-swollen films at equilibrium and in the dry state, respectively. 

The static water contact angles of the films were measured via a contact angle meter JGW-360B (Chengde chenghui testing machine co., LTD, Chengde, China). Approximately 5 μL of pure water was placed on the surface of the dried film. Thereafter, the shape of the drop was recorded within 5 s at 25 ± 1 °C. Ultra-pure water was used in these measurements.

## 3. Results

### 3.1. FTIR Characterization

Below, the result of FTIR characterization is reported. 

**Figure 1:**
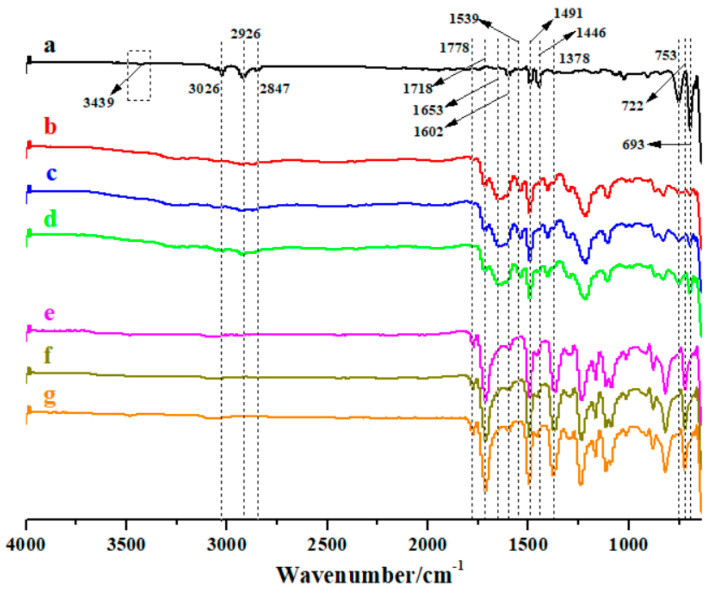
FTIR spectra of the synthesized polymers: (**a**), amino endcapped PS; (**b**), PAA_I_-*b*-PS; (**c**), PAA_II_-*b*-PS; (**d**), PAAI_II_-*b*-PS; (**e**–**g**), porous PI films from (**b**–**d**) by thermal treatment.

From the curve in Figure 1a, the absorption bands at 693 cm^−1^, 753 cm^−1^, 1446 cm^−1^, 1491 cm^−1^, 1602 cm^−1^, 2847 cm^−1^, 2926 cm^−1^ and 3026 cm^−1^ are characteristic peaks of polystyrene. The appearance of peaks at 3400~3500 cm^−1^ and 1602 cm^−1^ (overlapping with those of PS at ~1600 cm^−1^) suggests that the amino group was successfully endcapped to PS. In curves b-d, carbonyl absorption peak in -COOH, carbonyl absorption peak in -CONH and C-N stretching vibration peak in -CONH can be found at 1718 cm^−1^, 1653 cm^−1^ and 1539 cm^−1^, respectively; the aforementioned characteristic peaks of PS still exist, and the amino group disappears. Therefore, it is inferred that amino endcapped PS has been obviously introduced into the backbone of PAA. After thermal imidization as shown in curves e-f, the characteristic peaks of polyimide appear, in which the peaks at 1778 cm^−1^, 1718 cm^−1^, 1378 cm^−1^ and 722 cm^−1^ can be ascribed to asymmetric stretching vibration peak, symmetric stretching vibration peak, C-N stretching vibration peak and carbonyl absorption peak of imide ring, respectively; in the meantime, the characteristic peaks of PS disappear. This indicates that the pure PI forms after thermal treatment for PAA-*b*-PS.

### 3.2. Molecular Weight and Molecular Weight Distribution

**Figure 2:**
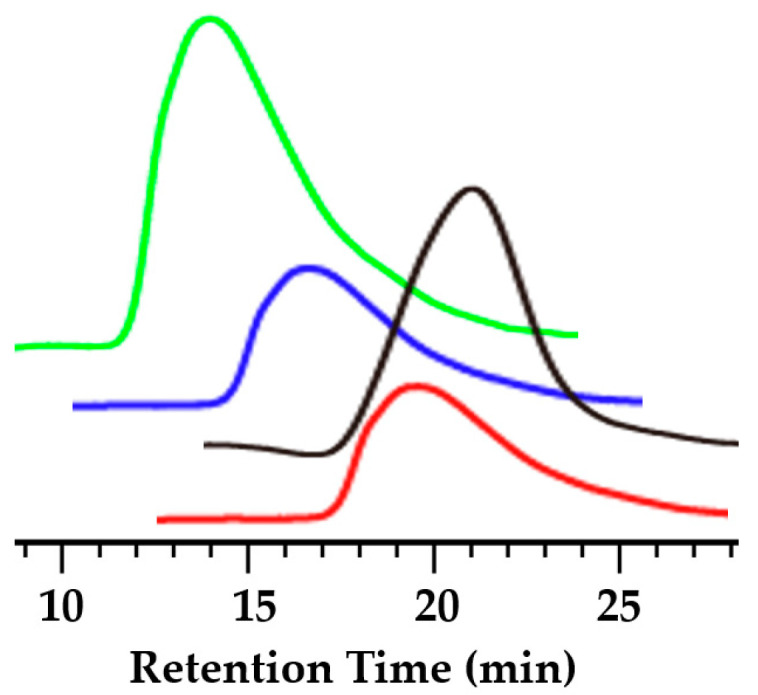
GPC curves of synthesized polymers: black, NH_2_-PS-NH_2_; green, PAA_I_-*b*-PS; blue, PAA_II_-*b*-PS; red, PAA_III_-*b*-PS.

Figure 2 shows the result of GPC characterization of synthesized polymers.

The characterization of synthesized polymers is summarized in Table 1.

### 3.3. TGA Characterization

**Figure 3:**
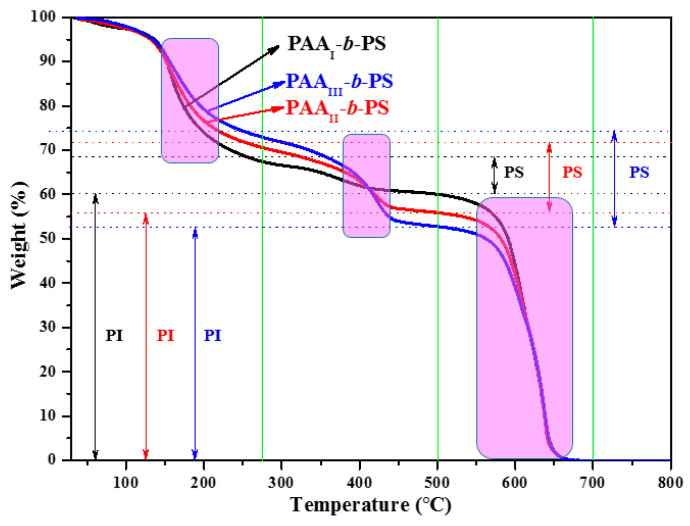
TGA curves of three block copolymers.

Figure 3 presents the TGA curves in a nitrogen atmosphere for PAA-b-PSs. 

The three transitions can be found in pink boxes from left to right: dehydration of PAA, decomposition of PS block and decomposition of PI block. After the transition of dehydration of PAA, PAA-*b*-PSs transform into PI-*b*-PSs. According to TGA curves, PI-*b*-PS and PI exist at 275 °C and 500 °C, respectively; no polymer exists at 700 °C. Therefore, *f ^V^_PS_* can be obtained, as shown in Table 2. 

### 3.4. Morphological Characterization

**Figure 4:**
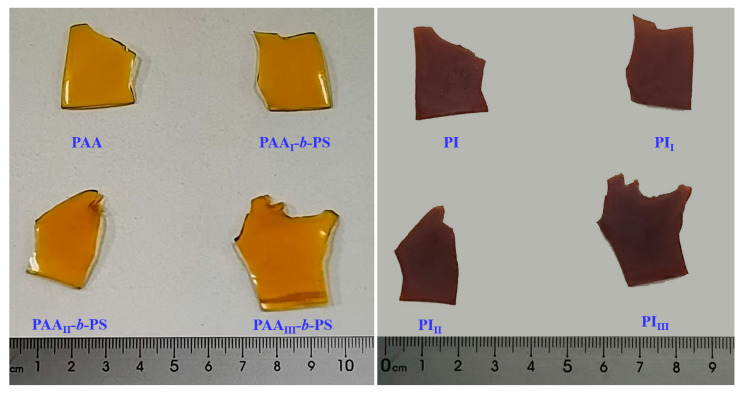
Appearance photographs of the films: **left**, PAA series films; **right**, PI films from thermal-treated PAA series films.

The appearance of films is shown in Figure 4.

The films turn from yellowish brown to deep brown after thermal treatment of pure PAA and PAA-*b*-PS films, as seen in Figure 4, and the size has a slight contraction after thermal treatment.

**Figure 5:**
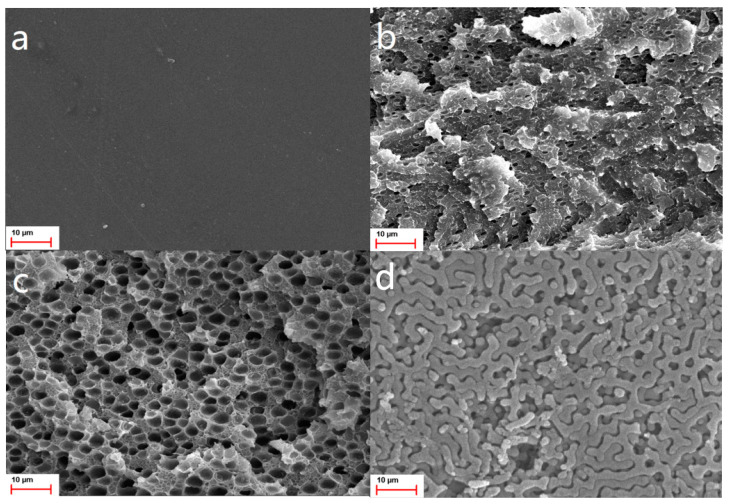
Cross-section SEM images of PI films from: (**a**), PAA by reaction of PMDA and ODA with equal mole ratio; (**b**), PAA_I_-*b*-PS; (**c**), PAA_II_-*b*-PS; (**d**), PAA_III_-*b*-PS.

Figure 5 shows the cross-section morphologies of PI films after 350 °C treatment.

It is normal that no pores appear in Figure 5a owing to the PS-free precursor of this PI film; there are a small number of sphere pores in PI films with *f ^V^_PS_* of 0.14 (Figure 5b), and the average pore diameter is approximately ~1.2 μm; widespread honey-combed pore morphology can be found with average pore diameter of ~3.5 μm in Figure 5c when *f ^V^_PS_* increases to 0.26, and many nanopores can be found amongst bigger pores. Interestingly, Figure 5d shows net-like porous channels with *f ^V^_PS_* increasing to 0.36. Obviously, porosity becomes higher with increasing *f ^V^_PS_*, as shown in Figure 5a,d. Basically, the formation of ordered microstructures from Figure 5b–d is considered as originating from self-assembly of PI-*b*-PS. Therefore, it is reasonable to speculate that the self-assembled micro-morphology of PI-*b*-PS can be finetuned by changing *f ^V^_PS_*.

### 3.5. Dielectric Properties

**Figure 6:**
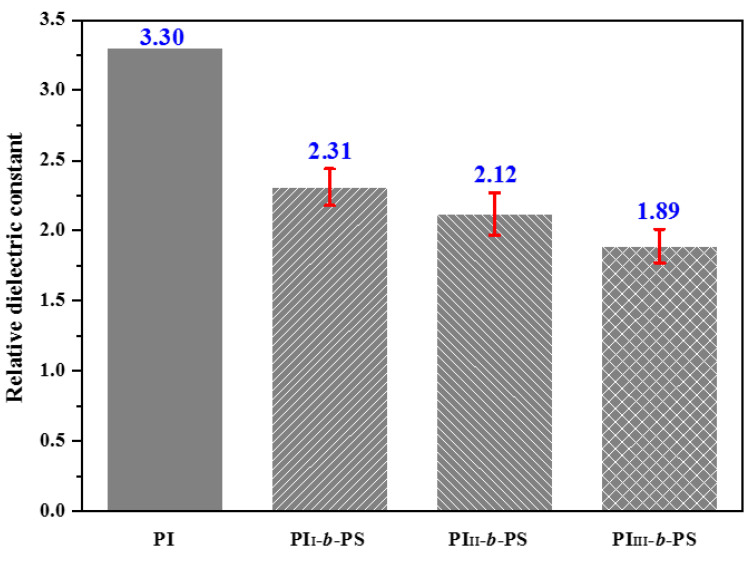
Relative dielectric constants (10^3^ Hz) of different PI films calculated by Equation (4).

The result of dielectric properties of films is displayed in Figure 6.

The pure PI film from PMDA and ODA has the relative dielectric constant of 3.30 (10^3^ Hz) [24]; when *f ^V^_PS_* increases, the relative dielectric constants of obtained PI films from PI-*b*-PS decrease as 2.31→2.12→1.89 (Figure 6) owing to increasing porosity (as seen from Figure 5). The relative dielectric constant of PI film derived from PAA-*b*-PSs can be controlled by changing the composition of degradable block. When the relative dielectric constant is 1.89, the porous PI film can be regarded as a low-κ material and has great potential for application in the field of microelectronics [26]. 

### 3.6. Liquid Uptake Behavior

**Figure 7:**
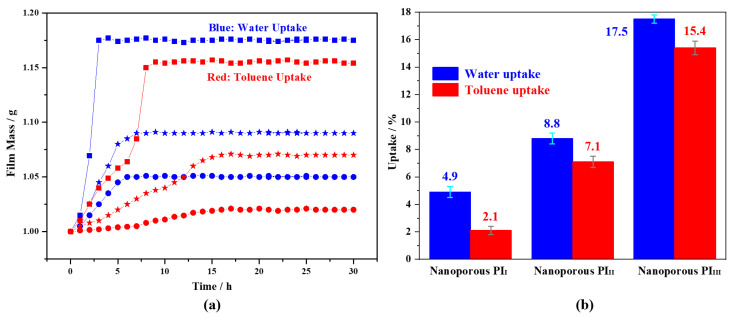
Uptake on water and toluene: (**a**), film mass–time curves, where sphere, star and square represent porous PI films (PI_I_, PI_II_ and PI_III_) from PI_I_-*b*-PS, PI_II_-*b*-PS and PI_III_-*b*-PS, respectively; (**b**), equilibrium uptake of porous PI films (PI_I_, PI_II_ and PI_III_) from PI_I_-*b*-PS, PI_II_-*b*-PS and PI_III_-*b*-PS.

The uptake performance of the prepared porous PI films is demonstrated in Figure 7.

The porous PI films have higher uptake of water and toluene with an increase in *f ^V^_P__S_* value (that is actually the porosity). Porous films of PI_I_ and PI_II_ (derived from PI_I_-*b*-PS and PI_II_-*b*-PS) reach equilibrium uptake of water in approximately 6 h with the final equilibrium uptake ratios of 4.9% and 8.8%, respectively, whereas porous film of PI_III_ (derived from PI_III_-*b*-PS) reaches equilibrium uptake of water in approximately 2.5 h with the final equilibrium uptake ratio of 17.5%, showing an amazing improvement of uptake. By contrast, porous films of PI_I_ and PI_II_ reach equilibrium uptake of toluene in approximately 15 h with the final equilibrium uptake ratios of 2.1% and 7.1%, respectively; porous film of PI_III_ reaches equilibrium uptake of toluene in about 8 h with the final equilibrium uptake ratio of 15.4%, indicating a remarkable promotion of uptake compared with those of porous films of PI_I_ and PI_II_. 

Accordingly, the uptake of porous PI film to water is higher than that to toluene at the same porosity, which is related to numerous imide and ether bonds in PI backbone and is naturally more beneficial to water affinity than to toluene affinity; porous PI film with porosity of 0.36 (with a net-like microstructure) can reach the final equilibrium uptake ratio higher with less time, compared with porous PI films with porosity of 0.14 and 0.26, which indicates that solvent uptake performance associates not only with porosity but also with the self-assembled microstructure of PI-*b*-PS. It is most likely that this net-like porous microstructure has interconnecting channels, which is essentially favorable for liquid uptake, resulting in higher uptake and reaching equilibrium uptake state faster, whether for water or for toluene. The materials with net-like the ordered porous microstructure have better performance than those with other porous microstructures, as reported elsewhere [27,28].

### 3.7. Water Contact Angle

**Figure 8:**
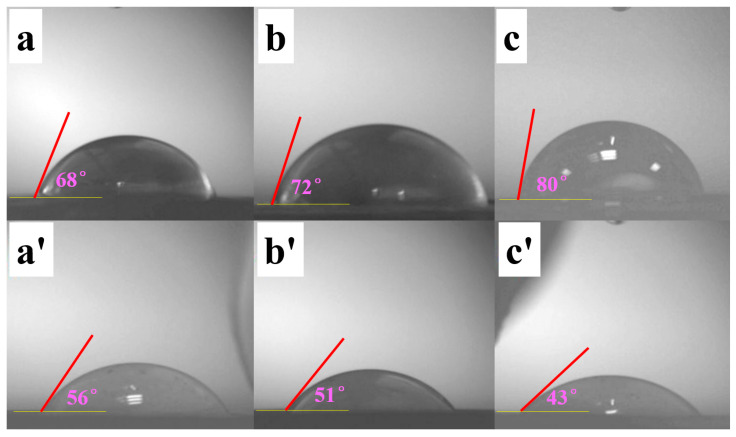
Contact angles of films: (**a**–**c**) represent PAA_I_-*b*-PS, PAA_II_-*b*-PS and PAA_III_-*b*-PS, respectively; (**a’**–**c’**) represent porous PI from PAA_I_-*b*-PS, PAA_II_-*b*-PS and PAA_III_-*b*-PS, respectively.

Figure 8 reflects the change of the water contact angles of the fabricated films.

When the volume fraction of PS increases, the contact angles change to 68°, 72° and 80° for the films of PAA_I_-*b*-PS, PAA_II_-*b*-PS and PAA_III_-*b*-PS, respectively, while the contact angles of the corresponding porous PI films decrease to 56°, 51° and 43°, respectively. It is concluded that PS is more hydrophobic compared with PAA derived from its lower polarity. Hence, in the PAA-*b*-PS series, it is normal that the film with higher content of PS has a higher water contact angle. Since PI from PMDA and ODA is also a polar polymer, a higher PS volume fraction in block copolymer indicates higher porosity when the PS block is removed along with the generation of porous PI. The remaining porous PI film with higher porosity has more air in its pores, indicating more favorability to be wetted by water. Consequently, the porous PI film with higher porosity has a smaller contact angle; namely, it has good wettability.

## 4. Discussion

### 4.1. Synthesis of PAA-b-PSs/PI-b-PSs

A series of PAA-*b*-PSs was synthesized successively via telomerization with polycondensation: telomerization was first conducted, in which PS were terminated at both ends by 4-aminophenyl groups from an amino terminated disulfide BAPD (Scheme 1), leading to the chain termini selectively reactive for subsequent polycondensation. Amino-endcapped NH_2_-PS-NH_2_, as a diamine, reacted with anhydride-endcapped PAA oligomers, which were prepared by PMDA and ODA in advance, forming PAA-*b*-PSs block copolymers. Here, diblock architecture PAA-*b*-PS appears in the repeat unit of “huge PAAs”. The synthesized PAA-*b*-PSs are not exactly an AB type of diblock copolymers, but an (AB)_m_ type of diblock copolymers (Scheme 2). 

The number-average and weight-average molecular weight as well as the molecular weight distribution (*Đ*) of PS block were determined by GPC. The *Đ* of PAA-*b*-PSs was determined by GPC. The number-average molecular weights of PAAs were determined by equation (2). Since PAA is the precursor of PI, it is eventually converted as PI after thermal imidization. On the basis of the molecular weight synthesized, the volume fraction of PS (*f ^V^_PS_*) was calculated by assuming that the densities of PS and PI are 1.02 [25] and 1.42 g/cm^3^ [24], respectively. The characterizations of synthesized PAA-*b*-PSs/ PI-*b*-PSs are summarized in Figure 1, Figure 2, Table 1 and Table 2.

### 4.2. Fabrication of Ordered PI Films from Self-Assembly of PAA-b-PSs

As is known, block copolymers can self-assemble into ordered nano-morphologies, such as sphere, cylinder, network and lamella, depending on different constituted compositions and molecular weights [29]. The solubility parameters of PS, PAA and PI are 18.6 (J/cm^3^)^1/2^, 31.9 (J/cm^3^)^1/2^, 35.6 (J/cm^3^)^1/2^, respectively [30,31]; microphase separation of PAA-*b*-PS and PI-*b*-PS happens very easily owing to the evident differences (i.e., chemical incompatibility) of PS vs. PAA and PS vs. PI. 

The PAA-*b*-PS solution of DMAc was casted on a glass substrate. When DMAc was removed at 50 °C for 8 h under vacuum, the PAA-*b*-PS film was thermally treated after the programmed temperature. PI generates accompanied by the decomposition of PS, suggesting porous structure generation in the PI matrix. DMAc has a boiling point of 164 °C, and DMAc removal at 50 °C under normal air pressure takes much longer time compared with the vacuum condition. Therefore, during this fabrication process, that the PAA-b-PS solution of DMAc was dried at 50 °C for 8 h under vacuum can make PAA block and PS block has enough time (neither too long time, nor too short time) to rearrange to microphase separate and self-assembly into ordered microstructure. This is based on the reason that a decrease in temperature can increase Flory–Huggins parameter χ (specifies the degree of incompatibility between the two blocks of a diblock copolymer), which has more tendency to microphase separate because χ is found to be inversely proportional to temperature [17]. 

For PAA from PMDA and ODA, the molecular weight of the repeat unit is 418; that value changes to 382 after PAA transforms into PI. Thus, *f ^V^_PS_* remains nearly unchanged when PAA-*b*-PS transforms into PI-*b*-PS. It is deduced that the self-assembled morphologies of PAA-*b*-PS are nearly similar to those of PI-*b*-PS. 

The dried PAA-*b*-PS films were thermally treated under a flowing nitrogen atmosphere in a sequential procedure of 100 °C 1 h + 200 °C for 1 h and 350 °C for 4 h with a mild heating rate. At 100 °C, PS is near its glass transition temperature, and polymer chains possess enough mobility to release the trapped trace amount of DMAc. There is no concern that the ordered microstructure of PAA-*b*-PS formed can be destroyed into a disordered microstructure at 200 and 350 °C. Because PAA and PI have a very high glass transition temperature (much higher than 350 °C for PMDA and ODA type PI), they are still in a glass state with rigid polymer chains, and hence, the formed ordered microstructure at 50 °C can be preserved well until PS is decomposed completely at 350 °C. Finally, porous PI films are formed as shown in Figure 5. 

Structurally, there exists a strong intermolecular and intramolecular interaction in the conjugated aromatic structures of PI mainchain, making it easy to form a charge-transfer complexing (CTC) between alternating electron–donor (diamine) and electron–acceptor (dianhydride) segments [32]. The stronger the electron-donating capability of the diamine residual group and the stronger the electron-accepting capability of the dianhydride residual group, the greater the degree of charge-transfer complex formation, and the easier light is absorbed. Naturally, color becomes darker with more absorption. The electron-donating capability of the diamine residual group and the electron-accepting capability of the dianhydride residual group in PAA mainchain are weaker than those of PI mainchain, resulting in pure PAA and PAA-*b*-PS films turning from yellowish brown to deep brown after thermal treatment (see Figure 4); dehydration of PAA and decomposition of PS block during thermal imidization cause the slight contraction of size when comparing with PI, as found by other researchers [20].

Sphere, honeycomb and net-like pores appear in SEM images in Figure 5, corresponding to *f ^V^_PS_* values of 0.14, 0.26 and 0.36, respectively. Honeycomb pores in Figure 5c look very much like close-packed hexagonal structure; net-like pores are very probably an ordered microstructure, which needs to be further identified by other more accurate characterization. Though these pores are at micrometer-sized level, these three ordered pores corresponding to different *f ^V^_PS_* values are highly consistent with the morphologies in general self-assembly of diblock copolymers in reference [29]. There is still a need to address whether the (AB)_m_ type of diblock copolymers in this article results in self-assembly at a micrometer-sized level or other influence factors cause this phenomenon.

According to the above analysis, PAA-*b*-PS can self-assemble into different morphologies, though it is an (AB)_m_ type of diblock copolymer by which ordered porous PI films generate.

### 4.3. Performance of Ordered Porous PI Films from PAA-b-PSs

The acquired ordered porous PI films maintain excellent high-temperature resistance evidenced by TGA analysis (Figure 3), in which the initial thermal decomposition is higher than 450 °C. 

The performances of porous PI films change as porosity (i.e., *f ^V^_PS_* of PAA-*b*-PS) changes: liquid uptake ability improves as *f ^V^_PS_* increases; relative dielectric constant and water contact angle become lower as *f ^V^_PS_* decreases. Particularly, the net-like pore structure in Figure 5d displays interconnecting porous channels, which is very important for the performance enhancement of porous PI films. Firstly, this pore structure has a higher surface area, and porosity is only determined from the SEM image, contributing to a lower relative dielectric constant (Figure 6); secondly, it is deduced that the interconnecting porous channels originate from through-hole and continuous structures, which is very helpful for liquid uptake depending on capillary force, and absorbed liquid can fill the porous channels thoroughly. Consequently, porous PI_III_ film achieves maximum liquid uptake in minimum time.

## 5. Conclusions

Similar to pure AB type diblock copolymer, the (AB)_m_ type of diblock copolymer PAA-*b*-PS/ PI-*b*-PS can also self-assemble into ordered microstructures that can be used as templates to fabricate ordered porous PI films. The characterization of the ordered porous PI films derived from PI-*b*-PS diblock copolymer was carried out, focusing on the effect of PS content. Changing PS content can finetune the self-assembled microstructures of PI-*b*-PS and can then regulate properties of the prepared porous PI films: solvent uptake ability can be improved by increasing PS content; relative dielectric constant and water contact angle become lower as PS content decreases. Porous structures from net-like morphology seem more favorable to enhance the properties of porous materials. In-depth research is being carried out to evaluate the specific relationship between microstructure and performance with further investigation of more microstructured porous PI films.

## Figures and Tables

**Table 1:** Characterization of synthesized polymers.

Code	*^a^*$\bar{M_{n}}$ (g/mol)	*^a^ Đ*	*^b^*$\bar{M_{n}}$*_PAA_* (g/mol)	$\bar{M_{n}}$*_total_* (g/mol)
NH_2_-PS-NH_2_	9400	2.23	---	---
PAA_I_-*b*-PS	---	2.38	67,370	76,770
PAA_II_-*b*-PS	---	2.17	33,840	43,240
PAA_III_-*b*-PS	---	2.11	21,130	30,530

The data of column *a* and column *b* were calculated according to GPC and Equation (2), respectively.

**Table 2:** Volume fractions of PS block of PI-*b*-PS from PAA-*b*-PS.

Code	Ordinate Value at 275 °C	Ordinate Value at 500 °C	Ordinate Value at 700 °C	*^a^ f ^m^_PS_*	*^b^ f ^m^_PI_*	*^c^* Volume Fraction of PS Block (*f ^V^_PS_*)
PI_I_-*b*-PS	0.67	0.60	0	0.07	0.60	0.14
PI_II_-*b*-PS	0.70	0.56	0	0.14	0.56	0.26
PI_III_-*b*-PS	0.73	0.52	0	0.21	0.52	0.36

***^a^f ^m^_PS_*** and ***^b^f ^m^_PI_*** represent weight fractions of PS block and PI block, respectively; the data of column *c* were calculated by $f_{PS}^{V}=\frac{\frac{f^{m}{}_{PS}}{\rho_{PS}}}{\frac{f^{m}{}_{PS}}{\rho_{PS}}+\frac{f^{m}{}_{PI}}{\rho_{PI}}}$, in which *ρ_PS_* is 1.02 g/cm^3^ [25] and *ρ_PI_* is 1.42 g/cm^3^ [24].

## Data Availability

Not applicable.
